# OXITEST as a Screening Method to Evaluate Antioxidant Agents: A Study of Oxidative Stability of Fishmeal

**DOI:** 10.1155/ijfo/5537919

**Published:** 2025-04-21

**Authors:** Miguel Albrecht-Ruiz, Jordan Vito-Villa, Pedro Cueva Martínez, Alberto Salas-Maldonado

**Affiliations:** ^1^Biochemistry and Biopolymers Area, Research Direction-DIDITT, Technological Institute of Production, Callao, Peru; ^2^Callao Fishing and Aquaculture CITE (Center for Productive Innovation and Technology Transfer), Technological Institute of Production, Callao, Peru; ^3^Physicochemical Laboratory, Research Direction-DIDITT, Technological Institute of Production, Callao, Peru

## Abstract

Oxidation in fishmeal (FM) can generate sufficient heat to potentially trigger combustion, making it essential to assess its risk of self-ignition before transport to prevent fires. Ethoxyquin, commonly used to mitigate this risk, has been banned in some markets due to its genotoxicity, driving the search for alternative antioxidants. The traditional method to evaluate this risk, the SW-846 1050 test, is costly and time-consuming. We used the simpler and cheaper OXITEST method to assess the oxidative stability of FM by comparing oxygen consumption in FM with and without antioxidants. Fresh FM without antioxidants was used, which was stored at −30°C for 6 months. FM contained 7.2% moisture, 18.8% ash, 64.3% protein, and 9.7% crude fat; of the lipids, 75% were neutral, and 25% were phospholipids. The fatty acid profile of the lipids revealed high levels of EPA and DHA, with a DHA/EPA ratio greater than 1, which makes the FM more prone to oxidation compared to anchovy oil. Initial results demonstrated oxygen consumption in FM samples, although the inflection point (IP) was not detected. In a second step, after optimizing the sample volume (50 g), temperature (80°C), and time (4 h), oxygen consumption was evaluated by OXITEST in FM samples with increasing concentrations of ethoxyquin, demonstrating an inverse correlation between concentrations of ethoxyquin and oxygen consumption (Pearson's linear). Finally, we evaluated FM samples with various commercial antioxidants and compared the area under the curve for oxygen pressure versus time using FM alone as a negative control and FM with 750 ppm of ethoxyquin as the positive control. OXITEST measurements revealed differences in the rate of oxygen pressure loss among the studied agents, offering a comparative measure of antioxidant efficiency. The OXITEST method can be employed as a rapid and cost-effective method to evaluate oxidative stability and the effectiveness of antioxidants in FM.

## 1. Introduction

At present, global fishmeal (FM) production stands at approximately 5 million tons, with Peru being the primary supplier, accounting for 20% of the world's production. It is sourced mainly from Peruvian anchovy or “anchoveta” (*Engraulis ringens*). Between 2010 and 2022, its annual production averaged 1.02 million tons (https://www.indexmundi.com/agriculture/).

The industrial control of anchoveta capture for FM and fish oil (FO) production is synchronized with the spawning period. It operates during two capture seasons per year, preceding spawning peaks, during which an increase in crude fat content is observed in the entire specimen [1]. FM and FO are industrially obtained by pressing the wet mass of cooked fish [2]. The resulting product is dried and ground for FM production, while the pressing liquid is centrifuged to separate the oil. This oil originates from the accumulation of subcutaneous fat and primarily consists of neutral lipids (triglycerides [TGs]) [3]. Anchoveta FM typically contains 8%–10% crude fat, which is characterized by a high content of omega-3 polyunsaturated fatty acids (*Ω*-3 PUFAs), primarily eicosapentaenoic acid (EPA, C20:5 *Ω*-3) and docosahexaenoic acid (DHA, C22:6 *Ω*-3) [4]. During FM production, these *Ω*-3 PUFAs undergo oxidative processes throughout stages such as cooking, drying, and grinding [5]. The PUFAs present in the final product remain highly susceptible to further oxidation, leading to heat release and increasing the risk of self-combustion and ignition. Therefore, effective management practices are crucial to minimize the risk of ignition during transport, especially considering the prolonged storage times in cargo holds of ships within supply chains [6].

The FM industry addressed this issue by incorporating antioxidants, inhibiting the exothermic oxidative reaction chain. These antioxidants encompass ethoxyquin, butylated hydroxytoluene (BHT), butylated hydroxyanisole (BHA), and certain tocopherols. Ethoxyquin proved to be the most efficient and serves as the standard against which the stabilizing effects of other antioxidants are measured [7–9]. After the manufacturing process, its addition marked a significant advancement for the FM industry, resulting in a product with regulated exothermic reactions while retaining its nutritional value (https://www.iffo.com/antioxidants-and-fishmeal). Consequently, FM fortified with antioxidants was included in the International Maritime Dangerous Goods (IMDG) Code, permitting relaxed stowage and ventilation requirements during maritime transport. It was categorized as a hazardous substance, Class 9: UN No. 2216 as amended 24-86 [10].

As the antioxidant in FM performs its function, it becomes depleted, underscoring the importance of its quantification. In the 1950s, the International Fishmeal and Fish Oil Organisation (IFFO) established the residual ethoxyquin concentration, setting recommended levels above the threshold necessary to prevent self-combustion, which are still accepted by some Asian countries. However, since 2019, the European Union has prohibited the use of ethoxyquin due to its potential genotoxicity [11, 12] and the detection of elevated levels of residual ethoxyquin in animals fed with such FM [13, 14]. Consequently, efforts are being made to identify alternative antioxidants capable of preventing self-combustion [15].

The assessment of FM self-combustion typically involves various methodologies, with [16] “Test C: Self-heating wastes,” being the most frequently employed. However, this method requires specialized equipment and extended execution time, making it impractical for a large number of studies on new FM antioxidants. Therefore, there is a need to develop new tests that are rapid and cost-effective, allowing screening for the selection of the most appropriate antioxidants for subsequent self-combustion assessments.

There are accelerated oxidation tests, with Rancimat and OXITEST being the most commonly used methods to measure the oxidative stability of food fats within a short timeframe, all without the need for expensive or toxic reagents [17]. The Rancimat (Metrohm, Switzerland) method assesses conductivity, which rises due to the concentration of volatiles dissolved in water from fat oxidized at high temperatures. This approach necessitates the sample pretreatment for the fat or oil extraction. The OXITEST (VELP Scientifica, Usmate [MI], Italy) method relies on recording oxygen pressure as samples exposed to high temperatures within sealed chambers consume oxygen. This results in a faster and more eco-friendly method [18] [19]. The OXITEST equipment measures the oxidative stability of raw materials and finished products without needing prior fat extraction, yielding satisfactory results for evaluating food products [20] [21]. The equipment's response is the inflection point (IP), which indicates a “stability time” before fat oxidation and corresponds to the pressure drop of O_2_ due to its consumption by the sample.

This study aimed to utilize the OXITEST equipment to measure the oxidative stability of FM treated with various antioxidants, comparing it with FM containing ethoxyquin (Control+) and with FM without antioxidants. This was conducted to establish a screening method that allows for the rapid and cost-effective selection of the most appropriate antioxidants for the search for new alternatives to be used in FM.

## 2. Material and Methods

### 2.1. Raw Material

The freshly produced anchoveta FM, without added antioxidants, was portioned into polyethylene bags of approximately 1 kg, vacuum sealed, and stored at −30°C until analysis. Additionally, FO was also sampled at an approximate date. The raw material was supplied by Pesquera Diamante S.A.C.

### 2.2. Physicochemical Tests

Moisture was determined by oven drying at 101°C using a Memmert UE 400 oven until constant weight [22]. The ash content was determined in a muffle furnace at 500°C (Barnstead/Thermolyne 48000) until constant weight [23]. Crude protein was determined by the Kjeldahl method using a Kjeldatherm/Turbosog/Vapodest system (C. Gerhardt GmbH & Co. KG) with a total nitrogen (TN) to protein conversion factor of 6.25 [24]. Crude fat was obtained using hexane as an extraction solvent with Soxtherm equipment (C. Gerhardt GmbH & Co. KG) [25].

To analyze the lipid composition, lipids were extracted from fresh FM (LT) using the Bligh and Dyer methodology [26], separating the neutral fraction, rich in TG, from the polar fraction, rich in phospholipids (PLs). Additionally, total lipids were extracted from oxidized FM (LTox). The TG and PL fractions were obtained by agitating 2 g of LT in 50 mL of acetone for 90 min, followed by a 16-h resting period at 4°C [27]. The resulting precipitate corresponded to the polar lipid-rich fraction (PL), while the soluble phase contained primarily neutral lipids, predominantly TG.

The fatty acid percentage profile of each fraction, as well as that of anchovy oil (FO) for comparative purposes, was determined by gas chromatography (GC). For this analysis, lipids were hydrolyzed with 2 N NaOH in methanol and subsequently esterified in a 2 N HCl methanol medium to obtain fatty acid methyl esters (FAMEs), following the method described by Kyriakidis and Dionysopoulos [28]. FAMEs were identified by comparing their retention times with those of a marine-derived standard mixture using a PerkinElmer Autosystem XL GC System gas chromatograph. The system was equipped with a SUPELCOWAX 10 column (30 m × 0.32 mm I.D., 0.25 *μ*m film thickness) and a flame ionization detector. Injection conditions were set at 250°C, with a detector temperature of 270°C. The oven temperature program started at 170°C, increased at a rate of 1°C/min, and reached a final temperature of 220°C. All analyses were performed in duplicate, and results are expressed as the percentage (%) of total fatty acids.

### 2.3. Sample Preparation for OXITEST Evaluation

Each bag of FM was tempered at 25°C for 2 h, placed on a tray, and sprayed with the antioxidant diluted in 30 mL of ethyl ether. The FM was mixed for 25–30 min using a sieve with a 1 mm mesh diameter to ensure the homogeneous distribution of the antioxidant and the evaporation of ethyl ether. This mixture was then placed in the equipment for evaluation.

### 2.4. Oxidative Stability Analysis

The analysis was conducted using the OXITEST, which comprises two thermostated and hermetically sealed titanium chambers, each capable of accommodating up to three titanium sample holders. It is coupled with a PC running OXISoft software for data processing (VELP Scientifica, Usmate, Italy). The OXITEST measures the drop-in pressure in the oxidation chamber resulting from the sample oxygen consumption, with its response being the IP, which OXISoft graphically estimates.

### 2.5. Initial Tests

Initial tests were conducted to establish working conditions, including the quantity of FM and temperature. The equipment manual suggests varying sample quantities based on the content of PUFAs present while also recommending working temperatures not exceeding 90°C to avoid interference from lipid autoxidation. Random tests were conducted with varying weights and temperatures, at an initial oxygen pressure of 6 bar, monitored for 12 h. All sample holders were used in each reactor chamber. The results revealed some peculiarities, prompting data extraction from the software for analysis, which was then transferred to an Excel sheet.

Additionally, tests were conducted using fresh anchovy oil (FO) and anchovy meal oxidized by heating at 90°C for 12 h (FM^ox-12h^) to compare and verify the OXITEST response.

### 2.6. Ethoxyquin FM Evaluation

To evaluate the relationship between oxygen consumption (measured by pressure) and antioxidant activity, ethoxyquin was added to FM at concentrations of 0, 50, 100, 350, and 750 mg/kg. These samples were then analyzed using the OXITEST. The 750 mg/kg concentration of ethoxyquin, commonly used in FM production, served as the Control+, while the FM without antioxidants only received 30 mL of ethyl ether. The test conditions were 50 g of sample, 80°C for 4 h, with an initial oxygen pressure of 6 bar.

### 2.7. Antioxidant Agents FM Evaluation

Twenty antioxidants available on the local market were evaluated, dissolved in 30 mL of ethyl ether in the quantities recommended by their data sheets. The antioxidants, both chemical and natural, included BHT, BHA, citrate, tocopherols, lecithin, propyl gallate, and rosemary extract, as well as mixtures of these. The response of each antioxidant agent evaluated was calculated by integrating the equation corresponding to each curve generated in the pressure versus time graph.

### 2.8. Statistics

The proximate characterization of FM and its lipid content, as well as the initial tests of the OXITEST response in FO, were performed in duplicate. Subsequent OXITEST measurements were carried out in triplicate. To assess the correlation between the ethoxyquin concentration and oxygen consumption (measured as pressure loss), Pearson's correlation was used. For the correlation study, principal component analysis (PCA) was carried out in order to determine the interrelationships between the response variables (final pressure, area, and efficiency) and the different antioxidants evaluated. PCA plots were produced using the statistical software Statistica, Version 12.

## 3. Results and Discussion

### 3.1. Physicochemical Tests

**Figure 1:**
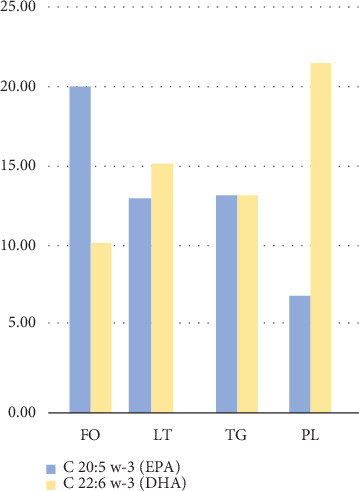
EPA and DHA contents in FO, fresh fishmeal lipids without antioxidant (LT), triglyceride-rich fraction (TG), and phospholipid-rich fraction (PL) of lipids obtained from fishmeal.

FM contained 7.2% moisture, 18.8% ash, 64.3% protein, and 9.7% crude fat. The lipid content extracted using the Bligh and Dyer method (LT) was 9.3%. Within LT, the PL-rich fraction accounted for 21.6%, and the neutral lipid-rich fraction (TG) represented 73.6%, which are values consistent with those reported in the literature [29]. The fatty acid profile of FO and LT showed high percentages of EPA and DHA (Table 1), which are characteristics of anchovy. In FO, the EPA content was higher than DHA, whereas in the FM lipids (LT), DHA predominated, particularly in the PL-rich fraction (Figure 1) [30, 31].

FO is primarily composed of neutral lipids and displays a DHA/EPA ratio of < 1. This pattern was similarly observed in historical data collected between 1997 and 2000 [32], where the DHA/EPA ratio ranged from 0.50 to 0.65. In contrast, FM, which contains a higher proportion of structural lipids (mainly PL), exhibits a DHA/EPA ratio of > 1, similar to values found in dark and light muscle tissue of anchovy [33], with ratios ranging from 1.34 to 2.42, and those reported in lipids extracted from whole very fresh anchoveta [34] that showed a ratio of 2.13. In our study, the DHA/EPA ratio in FO was 0.53, while in LT, it was 1.11. This suggests that the DHA content in FM is rapidly impacted during processing, leading to a decrease in the PUFA percentage, though maintaining a sufficient concentration to sustain ongoing oxidation. High-temperature treatments under oxidative conditions significantly reduce the PUFA content but do not completely eliminate it, as indicated by the lipid profile of oxidized FM (LTox in Table 1).

It is important to note that FM is a dry, ground mixture composed of muscle, heads, viscera, and backbones [35]. Consequently, its fat content includes a considerable proportion of structural lipids, mainly PL. These structural lipids are of interest due to their enhanced bioavailability and greater resistance to oxidation compared to TG. However, the proportion and distribution of PUFAs within the glycerol backbone remain a topic of ongoing discussion [36]. In our study, the high PUFA content in FM lipids likely increased their susceptibility to oxidation, especially when subjected to elevated cooking and drying temperatures.

### 3.2. Initial Tests

**Figure 2:**
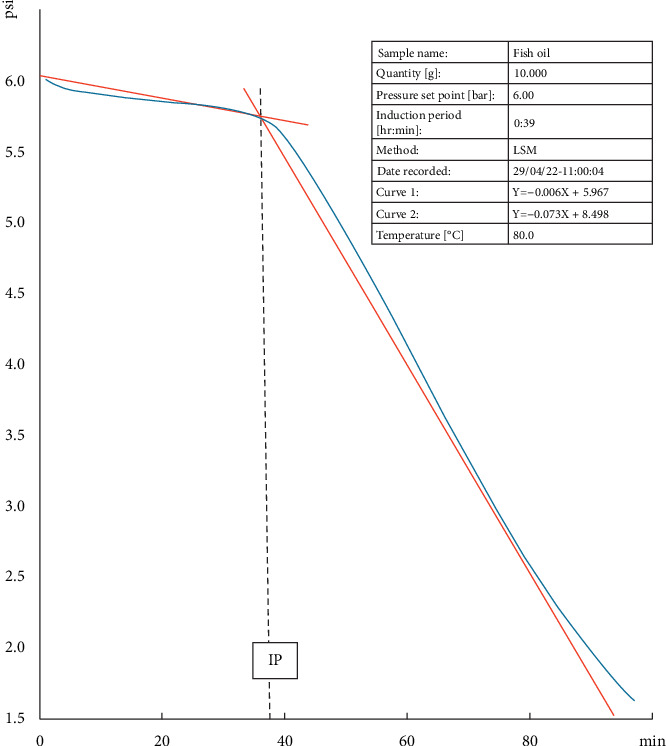
Accelerated oxidation of 10 g of anchovy oil (FO) at 80°C revealed an IP in the OXITEST response at 38 min, determined by the intersection of tangent lines formed by the graph. The FO was analyzed 24 h after the extraction.

To assess the response of the OXITEST to samples with a high PUFA content, 10 g of fresh FO was introduced into the oxidation chambers and exposed to a temperature of 80°C. The equipment automatically detected the IP at 38 min, accompanied by a curve consistent with the expected behaviour for nonoxidized fatty products (Figure 2). The oxygen pressure monitoring depicted in Figure 2 illustrates the curve's inflexion coinciding with the IP. The IP is defined as the time required to initiate oxidation cascade, indicating a sudden change in the oxidation rate. A longer IP means more stability against oxidation.

**Figure 3:**
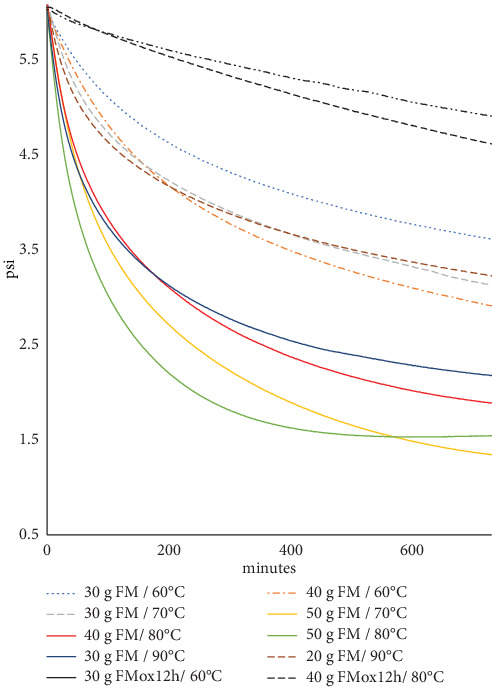
Pressure curves given during treatment of FM and FM^ox-12h^ in chambers OXITEST under different conditions of weights and temperatures.

Subsequently, to determine the appropriate amount of FM in the OXITEST, between 30 and 50 g of FM were randomly placed in the chambers that were subjected to temperatures between 60°C and 90°C. The OXITEST response displayed graphs different from what was anticipated and did not indicate the IP. However, oxygen consumption was evident (Figure 3). These graphs illustrated a more significant drop in oxygen pressure at the beginning of the measurement, followed by a gradual slowdown. The intersection of the lines was not discernible, and the IP values reported by the software varied without logical consistency. Consequently, the data was extracted from the OXISoft program and reanalyzed using a spreadsheet (Excel). Despite the anchovy meal containing sufficient concentrations of PUFA that consumed the oxygen in the chamber, the absence of IP suggested that oxidation cascade in the FM had commenced before being introduced into the reactor.

The oxygen pressure graphs (Figure 3) demonstrate that larger sample quantities provide more reactive sites for oxidation, leading to a more evident pressure drop (solid lines) compared to samples with smaller amounts (dotted lines). Conversely, the highly oxidized sample (FM^ox-12h^ in Table 1) exhibited minimal oxygen consumption (black dotted lines). The most pronounced pressure drop was observed when using 50 g of sample at 80°C during the first 4 h, which guided the selection of these conditions for the antioxidant evaluation.

### 3.3. Ethoxyquin FM Evaluation

**Figure 4:**
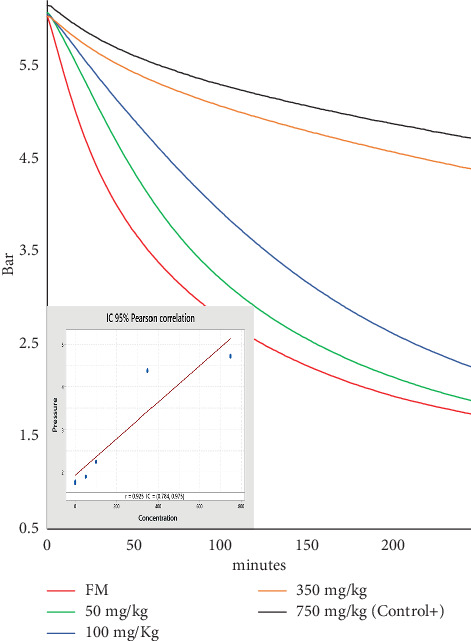
Pressure curves given by the OXITEST during the oxidation of FM with different concentrations of ethoxyquin and without antioxidant (FM). After 4 h, the Pearson correlation between oxygen pressure and ethoxyquin concentration yielded a strong positive correlation, with *r* = 0.9408.

Oxygen consumption in FM with increasing ethoxyquin concentrations was evaluated using the OXITEST. The graphs show an inverse correlation between ethoxyquin concentration and oxygen consumption (Figure 4), indicating that ethoxyquin partially reduces sample oxidation. After 4 h, the lowest oxygen consumption was observed in samples treated with 750 ppm of ethoxyquin (Control+). In contrast, the absence of antioxidants in FM resulted in higher oxygen consumption and a faster pressure drop.

The fatty acid profile of the lipids extracted from the Control+, after being subjected to OXITEST conditions, demonstrated ethoxyquin's effectiveness in protecting PUFAs. It preserved EPA and DHA concentrations at levels comparable to those in the total lipids (LT) from fresh raw material (Table 1), reaffirming its role as a reference antioxidant. Meanwhile, the FM subjected to OXITEST for 4 h (FM^ox-4h^) showed a significant reduction in the PUFA content, with DHA being the most affected due to its higher degree of unsaturation. Throughout the study, periodic measurements of FM and Control+ were performed to verify the equipment's consistency and response.

### 3.4. Antioxidant Agent FM Evaluation

The area under the oxygen pressure versus the time curve was determined for each assay. The areas obtained for each antioxidant were compared to that of Control+, considered 100%, and to that of FM, taken as 0%. The relative efficiency of each antioxidant was calculated using the following equation:

 $$\begin{array}{c} \text{Efficiency }\left(\%\right)=\frac{\text{Area}\left(\text{Control}+\right)-\text{area }\left(\text{FM}\right)}{\text{Area}\left(\text{antioxidant}\right)-\text{area }\left(\text{FM}\right)}*100 \end{array}$$

**Figure 5:**
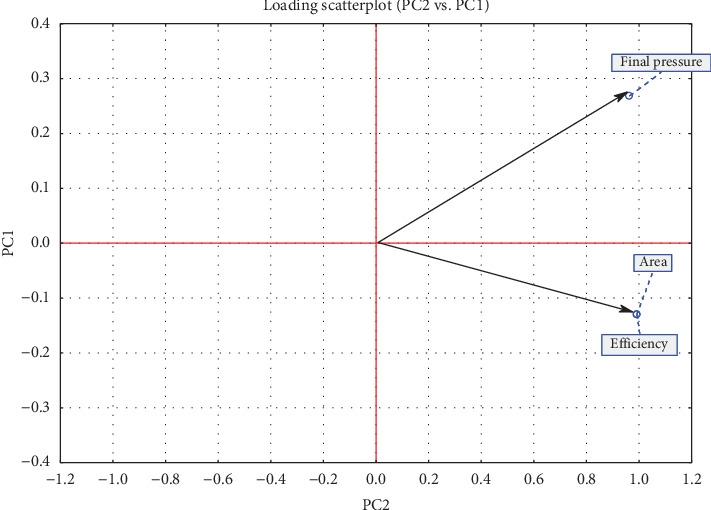
PCA loadings plot for three variables (final pressure, area and efficiency).

Different antioxidants at varying doses exhibited distinct responses (Table 2). PCA was performed on a scaled matrix comprising 32 treatments and three response variables (final pressure, area, and efficiency) to evaluate the influence of antioxidants at different concentrations. The analysis identified two principal components (PCs) with eigenvalues of 2.8939 and 0.1061, accounting for 96.46% and 3.54% of the total variance, respectively.  Figure 5 illustrates that the final pressure, area, and efficiency exhibit positive values and a strong correlation in both components.

**Figure 6:**
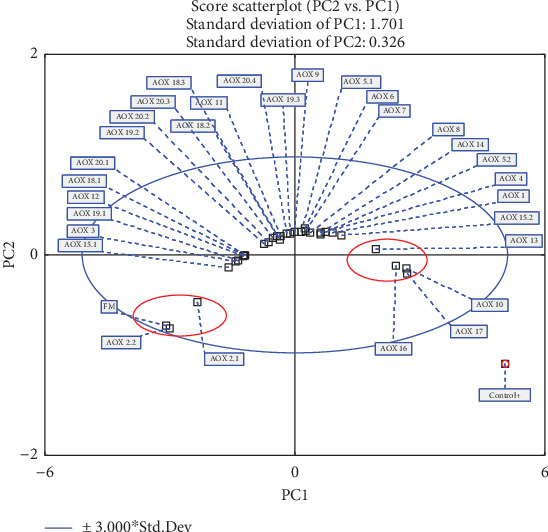
PCA scores plot for different antioxidants evaluated (32 samples).

The analysis of Figure 6 reveals the formation of three distinct groups based on the evaluated variables: final pressure, area, and efficiency. A blue ellipse representing the 95% confidence interval was applied to the PCA score plot, encompassing all samples within its boundaries. Most samples are located near the center of the PC1 and PC2 axes, while two well-defined groups, highlighted with red ellipses, stand out and are primarily represented by PC2. These groups correspond to FM, AOX 2.2, and AOX 2.1 (left side) and AOX 10, AOX 13, AOX 16, and AOX 17 (right side), which exhibit the lowest and highest values of the final pressure, area, and efficiency, respectively (Table 2). In this context, PCA indicates that AOX 10, AOX 13, AOX 16, and AOX 17 demonstrate the best performance in terms of oxidative stability compared to the other samples.

The concentration of the active principle should be considered in the future as these tend to lose their reactive capacity. Some antioxidant mixtures may be synergistic and enhance antioxidant capacity, while others may be inhibited. The present study will serve as an initial evaluation method for antioxidants in FM using the OXITEST to screen for those with better antioxidant capacity in a more economical and faster manner. Once the antioxidants with better effectiveness are chosen, they will be evaluated using already validated methodologies.

## 4. Conclusions

The OXITEST is a technique used to evaluate the oxidative stability of food products, characterized by the IP, which indicates the stability period before the onset of the lipid oxidation cascade. This stage is identified by a marked change in the O_2_ pressure drop within the reaction chambers. When anchovy oil was evaluated using the OXITEST at 80°C, the software reported an IP of 38 min. In contrast, when anchoveta FM was analyzed, an immediate pressure drop was observed without a clear IP, suggesting that the oxidation cascade in the FM lipids had already initiated, complicating the determination of the IP using this technique. Nevertheless, the observed pressure drops indicated that oxygen consumption was still ongoing. When 750 mg/kg of ethoxyquin was added to the FM, the rate of pressure decreases slowed down. As the ethoxyquin concentration was reduced, the pressure drop rate increased, with the antioxidant-free sample (FM) exhibiting the fastest decline in pressure.

The optimal conditions to observe pressure variations in the OXITEST were established at 50 g of FM at 80°C for 4 h. Several antioxidants available on the local market were tested, displaying different pressure reduction patterns, indicating variations in their capacity to inhibit oxidation. The efficiency of each antioxidant was quantified by calculating the area under the curve of their pressure versus time plots and comparing these areas to those obtained for the positive control (Control+) and the antioxidant-free sample (FM). Overall, it was observed that, in FM, the different antioxidants evaluated showed variable responses in terms of oxidative stability, where samples AOX 10, AOX 13, AOX 16, and AOX 17 provided greater oxidative stability.

This study suggests that the OXITEST method can be used as an effective tool to evaluate the efficiency of antioxidants in FM, providing a preliminary approach for identifying the most promising antioxidant compounds, which can later be subjected to more comprehensive validation studies.

## Figures and Tables

**Table 1:** Fatty acid profile (%) of fish oil (FO), lipids from oxidized fishmeal (LTox), and fresh fishmeal (FM). The total lipids from FM were extracted using the Bligh and Dyer method (LT), after which the triglyceride (TG) and phospholipid (PL) fractions were separated from LT.

**Fatty acids**	**FO**	**LT**	**TG**	**PL**	**FM** ^**ox-12h**^	**Control+**	**FM** ^**ox-4h**^
C 14:0 (myristic acid)	8.27	8.30	8.91	3.85	12.48	8.42	11.71
C 16:0 (palmitic acid)	16.85	21.56	21.26	25.01	31.92	21.46	29.12
C 16:1 (palmitoleic acid)	9.16	8.24	9.18	4.35	12.29	8.42	11.28
C 18:0 (stearic acid)	3.43	4.83	4.58	8.14	7.68	4.86	6.55
C 18:1 w-9 (oleic acid)	7.17	8.07	7.94	11.69	12.49	8.22	10.84
C 18:1 w-7 (vaccenic acid)	3.03	2.91	3.08	3.39	4.58	2.97	3.88
C 18:2 w-6 (linoleic acid)	0.81	1.62	1.77	1.05	1.66	1.66	1.76
C 20:5 w-3 (EPA)	**20.50**	**13.51**	**13.73**	**7.35**	**3.68**	**13.68**	**7.16**
C 22:5 w-3 (clupanodonic acid)	2.77	2.01	1.88	2.03	0.48	2.03	1.05
C 22:6 w-3 (DHA)	**10.77**	**15.72**	**13.76**	**22.11**	**2.26**	**14.91**	**5.67**
Total	82.73	86.77	86.06	88.95	89.49	86.63	89.02
Saturated	28.55	34.69	34.74	36.99	52.07	34.74	47.38
Monounsaturated	19.35	19.22	20.19	19.43	29.35	19.61	26.00
Polyunsaturated	34.84	32.86	31.13	32.53	8.07	32.28	15.64
EPA + DHA	**31.27**	**29.23**	**27.49**	**29.46**	**5.94**	**28.59**	**12.83**

**Table 2:** Evaluated antioxidants, doses, oxygen pressure in OXITEST chambers after 4 h, area under the curve generated by each antioxidant, and effectiveness compared to 750 ppm ethoxyquin (“Control+”). Data are presented as means (±standard deviations, *n* = 3). Different letters within the same treatment indicate statistically significant differences (*p* < 0.05) among the means.

**Treatment**	**Active principle**	**Doses (mg/kg)**	**Final pressure (bar)**	**Area (U2)**	**Efficiency (%)**
AOX 1	BHT crystalized	500	4.07 ± 0.01	2.57 ± 0.00	39.9

AOX 2	BHT aqueous dispersion	1240	1.81 ± 0.01a	0.68 ± 0.00a	1.2
		2000	2.26 ± 0.08b	0.96 ± 0.04b	6.9

AOX 3	BHT + BHA	1550	2.90 ± 0.01	1.33 ± 0.00	14.6

AOX 4	BHT + BHA+ Citrate	2493	4.01 ± 0.07	2.48 ± 0.08	37.9

AOX 5	BHT + BHA + citric acid	2810	3.76 ± 0.01c	2.13 ± 0.01c	30.9
		3750	3.94 ± 0.04d	2.42 ± 0.05d	36.8

AOX 6	BHT + BHA + citric acid	3333	3.82 ± 0.14	2.17 ± 0.13	31.7

AOX 7	BHT + BHA + citric acid	2632	3.81 ± 0.01	2.20 ± 0.04	32.2

AOX 8	BHT + propyl gallate	3000	3.85 ± 0.04	2.27 ± 0.05	33.7

AOX 9	BHT + BHA + propyl gallate	2917	3.69 ± 0.01	2.04 ± 0.00	29.1

AOX 10	BHT + BHA + citrate + propyl gallate	4170	4.61 ± 0.05	3.76 ± 0.05	64.1

AOX 11	BHT + BHA + citrate + propyl gallate	1745	3.52 ± 0.00	1.87 ± 0.00	25.5

AOX 12	Tocopherols	4444	3.01 ± 0.03	1.42 ± 0.02	16.5

AOX 13	Tocopherols + rosemary extract	2400	4.42 ± 0.00	3.24 ± 0.00	53.6

AOX 14	Tocopherols + rosemary extract	1579	3.95 ± 0.01	2.40 ± 0.01	36.4

AOX 15	Tocopherols + rosemary extract	2400	2.79 ± 0.02e	1.26 ± 0.01e	13.1
		4000	4.15 ± 0.01f	2.71 ± 0.03f	42.8

AOX 16	Tocopherols + propyl gallate + citrate	4000	4.52 ± 0.00	3.60 ± 0.03	60.8

AOX 17	Tocopherols + propyl gallate + citrate	2400	4.59 ± 0.03	3.79 ± 0.04	64.7

AOX 18	Mix tocopherols–soja oil	860	3.03 ± 0.03g	1.42 ± 0.02g	16.4
		1430	3.49 ± 0.02h	1.81 ± 0.00h	24.3
		2860	3.49 ± 0.11h	1.86 ± 0.12h	25.3

AOX 19	Tocopherols–soja oil	860	2.93 ± 0.09i	1.37 ± 0.06i	15.4
		1430	3.30 ± 0.08j	1.66 ± 0.07j	21.3
		2860	3.63 ± 0.03k	1.98 ± 0.00k	27.9

AOX 20	*α*-Tocopherol + starch modified	2069	3.04 ± 0.00l	1.44 ± 0.00l	16.8
		3448	3.36 ± 0.02m	1.71 ± 0.00m	22.3
		5813	3.43 ± 0.03m	1.76 ± 0.03m	23.3
		8621	3.60 ± 0.01n	1.94 ± 0.01n	27.0

Control+	Ethoxyquin 95%	750	5.03 ± 0.01	5.52 ± 0.12	100.0

FM	Without antioxidants	0	1.79 ± 0.04	0.62 ± 0.01	0.0

## Data Availability

The data used in this study, “OXITEST as a Screening Method to Evaluate Antioxidant Agents: A Study of Oxidative Stability of Fishmeal,” are available upon reasonable request from the corresponding author. This article has been prepared with full transparency regarding data management.
